# Clinical features and risk factors of HIV-infected patients with intracerebral hemorrhage: a retrospective study with propensity score matching analysis

**DOI:** 10.3389/fcimb.2024.1498327

**Published:** 2025-01-07

**Authors:** Qiuhui Huang, Shengri Chen, Hua Huang, Xuhui Deng, Gengyu Cen, Miao Wang, Zhijian Liang

**Affiliations:** ^1^ Department of Neurology, First Affiliated Hospital, Guangxi Medical University, Nanning, China; ^2^ Department of Neurology, The Affiliated Yuebei People’s Hospital of Shantou University Medical College, Shaoguan, China

**Keywords:** intracerebral hemorrhage, human immunodeficiency virus, risk factors, propensity score matching analysis, cerebrovascular disease

## Abstract

**Purpose:**

To investigate the clinical features and risk factors of the human immunodeficiency virus (HIV)-infected patients with intracerebral hemorrhage (ICH).

**Patients and methods:**

The patients with HIV-infected without ICH group were matched to the group of HIV-infected ICH patients. Logistic regression analysis using 1:1 propensity score matching (PSM) was performed to investigate the independent risk factors for ICH in HIV-infected patients. The receiver operating characteristic (ROC) curve was configured to calculate the optimal predictors of ICH in HIV-infected patients.

**Results:**

A total of 59 HIV-infected patients with ICH and 180 HIV-infected patients without ICH were included. A cohort of 118 patients was ascertained utilizing PSM. Multivariate binary logistic regression analysis revealed that drug abuse-related HIV-infected, prolonged prothrombin time (PT), and elevated triglyceride (TG) levels were independent risk factors of ICH in HIV-infected patients. The ROC curve demonstrated that the combined predictor, composed of drug abuse-related HIV-infected, prolonged PT, and elevated TG levels, exhibited the highest area under the curve (AUC), with a cut-off point at 0.426, sensitivity of 78%, and specificity of 81.4%.

**Conclusion:**

The present study revealed that a valuable factor combined with drug abuse-related HIV-infected, prolonged PT, and elevated serum TG levels could serve as predictors of ICH in HIV-infected patients.

## Highlights

- What is already known on this topic – Human immunodeficiency virus (HIV) -infected patients are closely related to cerebrovascular disease. Still, data on intracerebral hemorrhage (ICH) in HIV-infected patients remained limited.
- What this study adds – A valuable factor, combining drug abuse-related HIV-infected, prolonged prothrombin time, and elevated serum triglyceride levels, could serve as a predictor of ICH for HIV-infected patients.
- How this study might affect research, practice, or policy – The study’s findings assist clinicians in identifying HIV-infected patients with a heightened susceptibility to ICH, enabling them to implement efficacious interventions.

## Introduction

Human immunodeficiency virus (HIV) -infected patients are closely related to cerebrovascular disease (Cole et al., 2004). The incidence of ischemic stroke among HIV-infected patients has been found to increase and has received increasing attention (Ovbiagele and Nath, 2011; Abdallah et al., 2018; Jung et al., 2019; Schaefer et al., 2020). However, although the incidence of acute intracerebral hemorrhage (ICH) in HIV-infected patients was nearly equal to that of ischemic stroke (Cole et al., 2004), data on ICH in HIV-infected patients remained limited (Pinto AN., 1996). Patients with HIV-infected and ICH were observed in the early 1980s, and it was found that ICH tended to occur in patients in the later stages of HIV infection (Roquer et al., 1998). Moreover, it has been demonstrated that HIV infection increases the risk of ICH (Chow et al., 2014). A population-based study conducted in Central Maryland and Washington, D.C., in 2004 examined 171 patients with ICH, of whom six had HIV, and found an adjusted relative risk of 12.7 (95% CI, 4.0-40.0) for ICH, indicating a strong association between HIV infection and ICH (Cole et al., 2004). Recently, a systematic review and meta-analysis by Reza Behrouz in 2016, which included five studies from 1985 to 2010 and involved a total of 5,310,426 person-years, revealed that HIV-infected more than tripled the risk of ICH, particularly among those in advanced stages of the disease (Behrouz et al., 2016). Furthermore, a national retrospective study in the United States aimed at evaluating the prevalence of stroke among HIV-infected admissions found an increased overall prevalence of ICH among hospitalized individuals with HIV-infected (Patel et al., 2021). Additionally, ICH in HIV-infected patients was found to be associated with certain infections, such as neurosyphilis, toxoplasmosis, meningitis, cytomegalovirus encephalitis, and HCV, which can have significant neurological complications (Roquer et al., 1998; Durand et al., 2013; Patel et al., 2021). However, the clinical features and independent risk factors of ICH in HIV-infected patients remained not fully understood.

The present study aimed to investigate ICH’s clinical features and risk factors in HIV-infected patients using a retrospective propensity score matching (PSM) analysis. Eligible patients with HIV-infected ICH were included in the study. Meanwhile, age- and gender-matched HIV-infected patients without ICH, in a ratio of approximately 1:3, were included as controls. Univariate and multivariate binary logistic regression analyses were conducted to identify independent risk factors for ICH in HIV-infected patients, which should facilitate clinicians to identify HIV-infected patients who are at high risk for ICH and to implement adequate preventive measures.

## Materials and methods

### Patients

**Figure 1:**
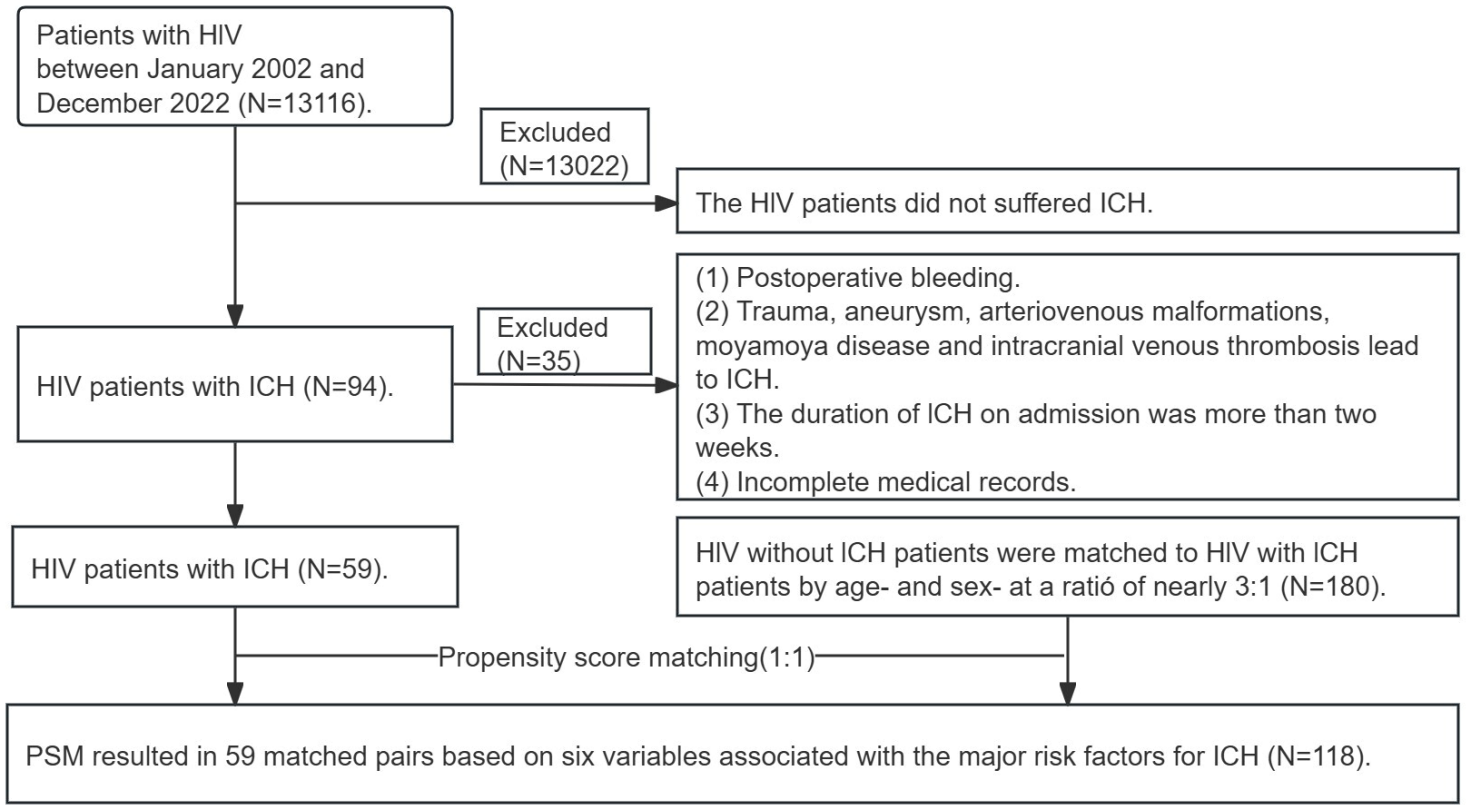
Flowchart of patient selection.

A retrospective analysis was conducted on the clinical records of HIV-infected patients who suffered ICH at the First Affiliated Hospital of Guangxi Medical University and the Fourth People’s Hospital of Nanning from January 2002 to December 2022. In the present study, ICH was diagnosed according to the 2022 American Heart Association/American Stroke Association (AHA/ASA) guidelines (Greenberg et al., 2022 2022) and confirmed by computed tomography (CT) or magnetic resonance imaging (MRI). The definition of HIV infection and its stages followed the Centers for Disease Control and Prevention (CDC) 2022 edition (Prevention CfDCa, 2022a; Prevention CfDCa, 2022b). Patients who met the following recruitment criteria were enrolled in the group of HIV-infected patients with ICH (HIV-infected ICH group): (1) age ≥ 18 years; (2) acute spontaneous ICH occurred within 2 weeks; (3) one of the HIV-infected tests (antibody tests, antigen/antibody tests, or nucleic acid tests) was positive. The exclusion criteria for the HIV-infected ICH group included: (1) postoperative bleeding, (2) ICH due to trauma, aneurysm, arteriovenous malformations, moyamoya diseases, or intracranial venous thrombosis, and (3) incomplete medical records. HIV-infected without ICH patients, who were age- and gender-matched to the ICH patients in a ratio of approximately 1:3, were enrolled in the control group (HIV-infected without ICH group). A retrieval flowchart is shown in **Figure 1**.

### Data collection

The clinical data of all patients included demographic characteristics, such as age, gender, the duration of HIV-infected, HAART usage, clinical manifestations, complications, and outcomes. The vascular risk factors included in our study, such as hypertension, hyperlipidemia, diabetes, alcohol consumption, and current smokers, were selected based on established guidelines and previous research (Greenberg et al., 2022 2022; Tu and Wang, 2023). Additionally, laboratory data were collected, including routine blood tests, coagulation function assessment, and determination of CD4^+^ and CD8^+^T cell counts. CT, MRI, computed tomography angiography (CTA), and other imaging examinations were performed. For the ICH group, laboratory and imaging data were collected within three days of admission. The diagnostic criteria of coagulopathy included platelet counts below 100/mm³, international normalized ratios (INR) exceeding 1.5, prothrombin times (PT) over 13 seconds, and activated partial thromboplastin times (APTT) exceeding 40 seconds, disseminated intravascular coagulation (DIC): fibrinogen<200 mg/dL and D-dimer>290 ng/dL. The diagnostic criteria for hypertriglyceridemia stipulated that triglyceride (TG) levels exceed 1.7 mmol/L. Accredited researchers reviewed electronic medical records and assessed the Modified Rankin Scale (mRS) scores at discharge (van Swieten et al., 1988). An mRS score of 0-2 indicated a favorable outcome, while a score of 3-6 denoted an unfavorable outcome.

### Statistical analysis

Statistical analysis was performed using IBM SPSS software (version 27.0). The Kolmogorov–Smirnov test assessed the normal distribution of variables. Variables with a normal distribution were presented as mean ± standard deviation, whereas non-normally distributed variables were expressed as median (25%-75%). Categorical variables were reported as counts (%). The Mann-Whitney U-test was used for non-normally distributed variables, whereas unpaired comparisons were conducted using Student’s unpaired t-test. Categorical variables were analyzed using Pearson’s χ^2^ or Fisher’s exact test.

PSM analysis was performed to mitigate the influence of conventional vascular risk factors and control for potential confounding factors to minimize bias. The variables considered for PSM, including age, gender, hypertension, diabetes, current smoking status, and alcohol consumption, were used. We established 59 matched pairs between the HIV-infected with the ICH group and the HIV-infected without the ICH group using a 1:1 nearest neighbor matching analysis.

Multivariate binary logistic regression analysis was used to identify independent risk factors of ICH in HIV-infected patients. The initial model inclusion criteria were univariate factors with *P* < 0.05. Subsequently, multivariate binary regression analysis was conducted with an entry and exit threshold value of 0.05. Model performance assessment involved analyzing the receiver operating characteristic ROC and incorporating the area under the curve (AUC), sensitivity, and specificity. The optimal threshold for identifying HIV-infected patients was determined by analyzing the receiver operating characteristic (ROC) curve and using the Youden index to establish the occurrence of ICH. Two-tailed *P* values were used, with statistical significance defined as *P* < 0.05. Given the unique characteristics of HIV-infected ICH patients, a formal sample size calculation was not performed. This research has received approval from the Ethics Committee of the First Affiliated Hospital of Guangxi Medical University.

## Results

### Patient profile

**Figure 2:**
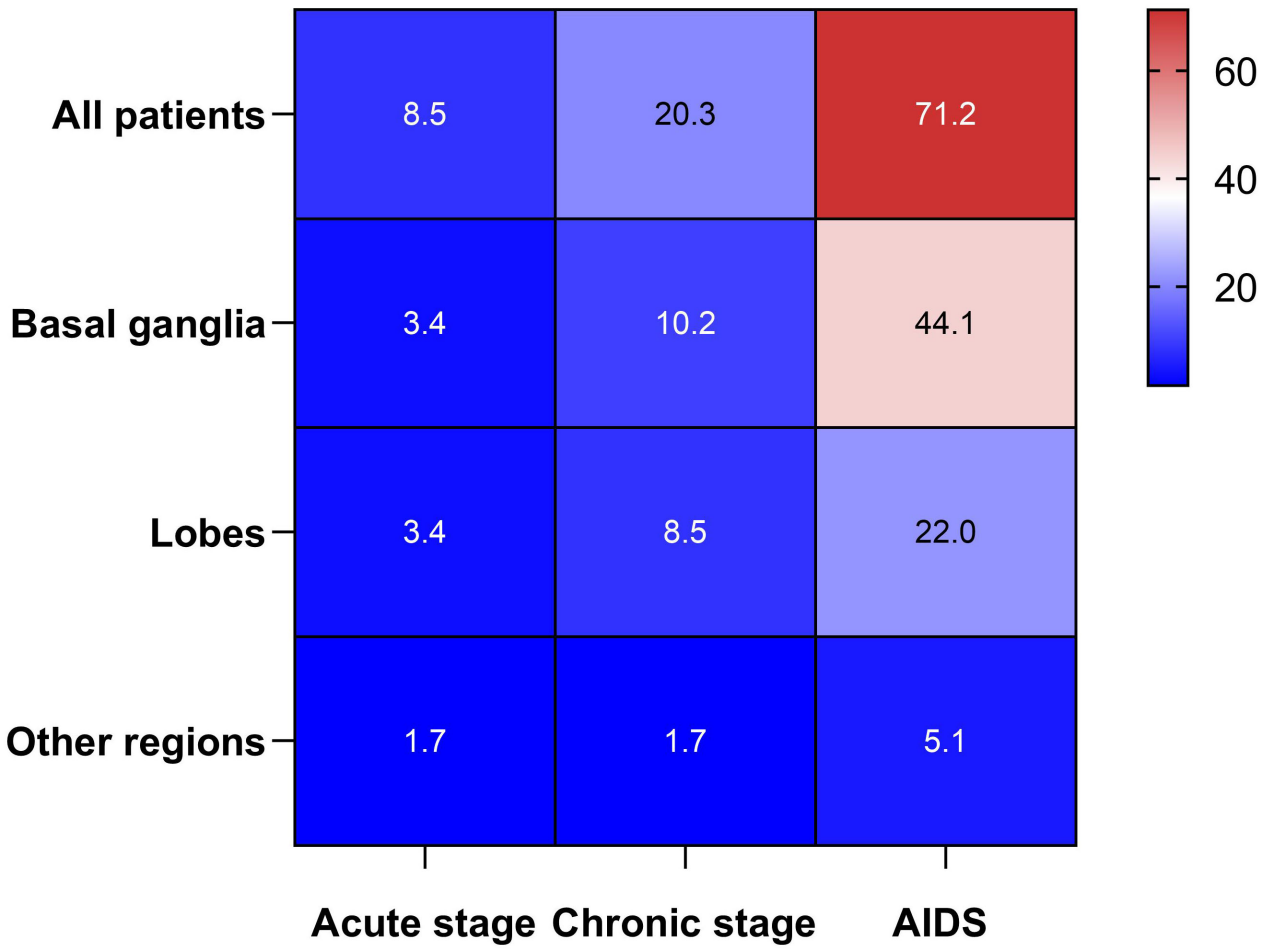
Heat map between the HIV-infected stage and the locations of ICH.

Of the 94 patients with ICH who were HIV-infected during the study period, 59 met the inclusion criteria and were included in the final analysis. Meanwhile, according to the present protocol, 180 patients with HIV-infected but without ICH were enrolled in the control group. Within the HIV-infected ICH group, there were 46 males (78.0%, 46/59) and 13 females (22.0%, 13/59), with a median age of 54 years (range: 47-67). Moreover, in the HIV-infected ICH group, 54 patients developed ICH after HIV-infected. In comparison, the remaining five patients had been hospitalized for their first-ever ICH before HIV-infected confirmed. Additionally, hemorrhagic lesions were most commonly found in the basal ganglia (57.6%, 34/59), followed by the lobes (33.9%, 20/59). Regarding the stages of HIV-infected, there were five patients in the early stage, 12 in the chronic stage, and 42 with acquired immunodeficiency syndrome (AIDS). Furthermore, patients with ICH who were HIV-infected commonly presented with symptoms such as limb weakness, consciousness disorder, and dizziness (**Table 1**, **Figure 2**). As a result of PSM, 118 patients were included in the study, creating an unbiased database with 59 matched pairs of participants.

**Table 1:** Demographics of HIV-infected ICH patients.

Characteristic	All patients, n = 59 (%)
Age	
18-45 (%)	10 (16.9)
45-60 (%)	28 (47.5)
>60 (%)	21 (35.6)
Male (%)	46 (78.0)
Vascular risk factors	
Hypertension (%)	30 (50.8)
Hypertriglyceridemia (%)	1 (1.7)
Diabetes (%)	6 (10.1)
Alcohol consumption (%)	14 (23.7)
Current smoker (%)	16 (27.1)
The interval between HIV-infected and ICH	
Before HIV infection confirmed (%)	5 (8.5)
After HIV infection (%)	54 (91.5)
Clinical manifestation	
Limb weakness (%)	29 (49.2)
Consciousness disorder (%)	25 (42.4)
Dizziness (%)	12 (20.3)
Headache (%)	11 (18.6)
Vomiting (%)	10 (16.8)
Dysarthria (%)	5 (8.5)
Paralysis (%)	5 (8.5)
Aphasia (%)	4 (6.8)
Epilepsy (%)	4 (6.8)
Delirium (%)	3 (5.1)
Nausea (%)	3 (5.1)
Numbness (%)	3 (8.5)
Locations of hemorrhagic lesions	
Basal ganglia (%)	34 (57.6)
Lobes (%)	20 (33.9)
Other regions (%)	5 (8.5)
The volume of hemorrhagic lesions	
<30 mL (%)	43 (72.8)
≥30 mL (%)	16 (27.1)
Stages of HIV-infected	
Acute stage (%)	5 (8.5)
Chronic stage (%)	12 (20.3)
AIDS (%)	42 (71.2)
HAART (%)	34 (57.6)

HIV, human immunodeficiency virus; ICH, intracerebral hemorrhage.

### Imaging features

**Figure 3:**
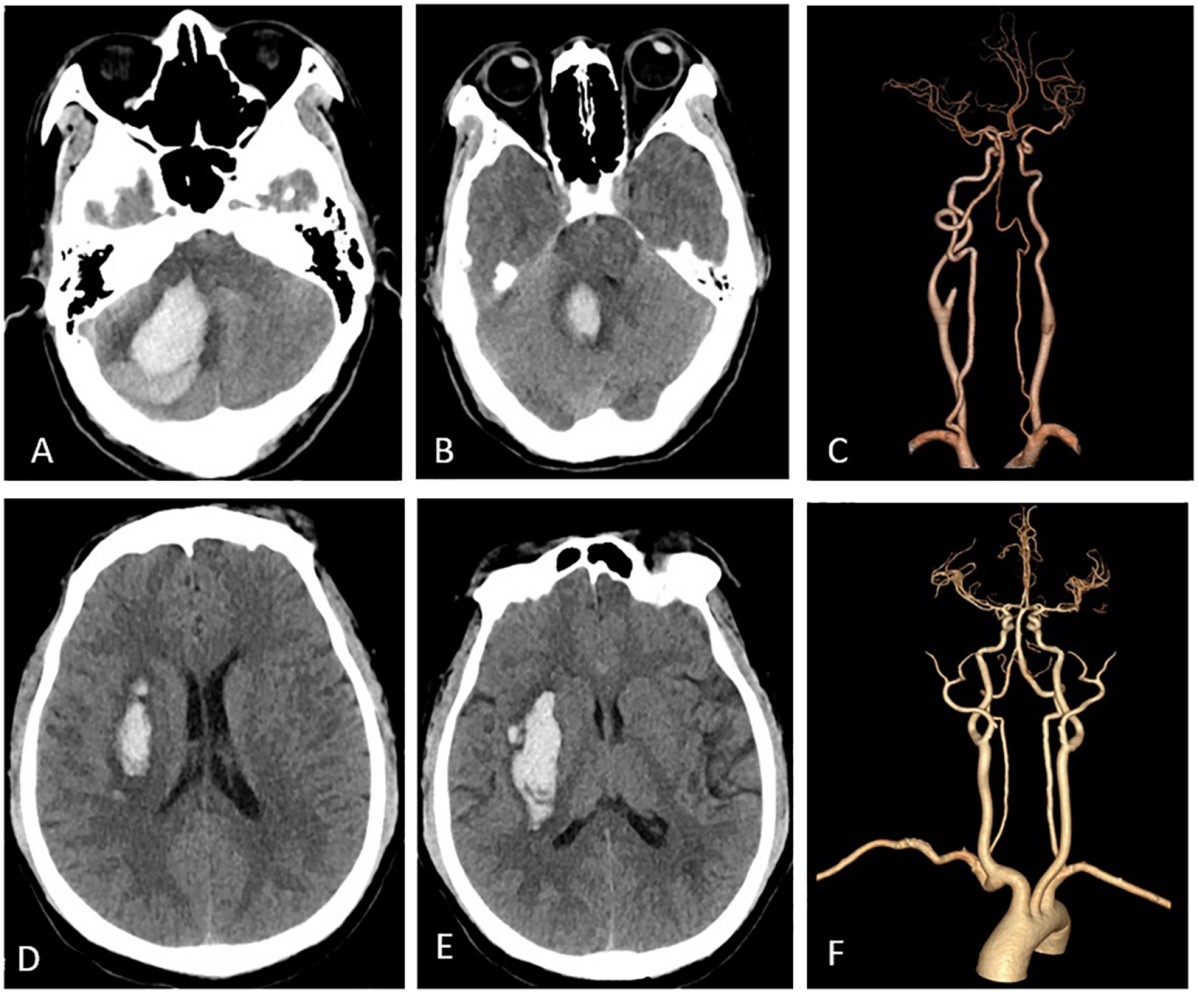
Imaging features. Panels **(A-C)** pertain to a 47-year-old female who was diagnosed with HIV-infected and subsequently developed a right cerebellar ICH. Panel **(A)** illustrates hemorrhage in the right cerebellar hemisphere, while in **(B)**, the ICH extends into the fourth ventricle, leading to its compression. Panel **(C)** depicts normal cerebral arteries. Panels **(D-F)** represent a middle-aged male patient with HIV-infected who experienced ICH. Specifically, **(C, D)** reveals bleeding in the right basal ganglia, while **(F)** displays normal cerebral arteries.

CT and CTA revealed the characteristic imaging features of ICH in patients with HIV infection (**Figure 3**).

### The outcome of ICH in HIV-infected patients at discharge

**Figure 4:**
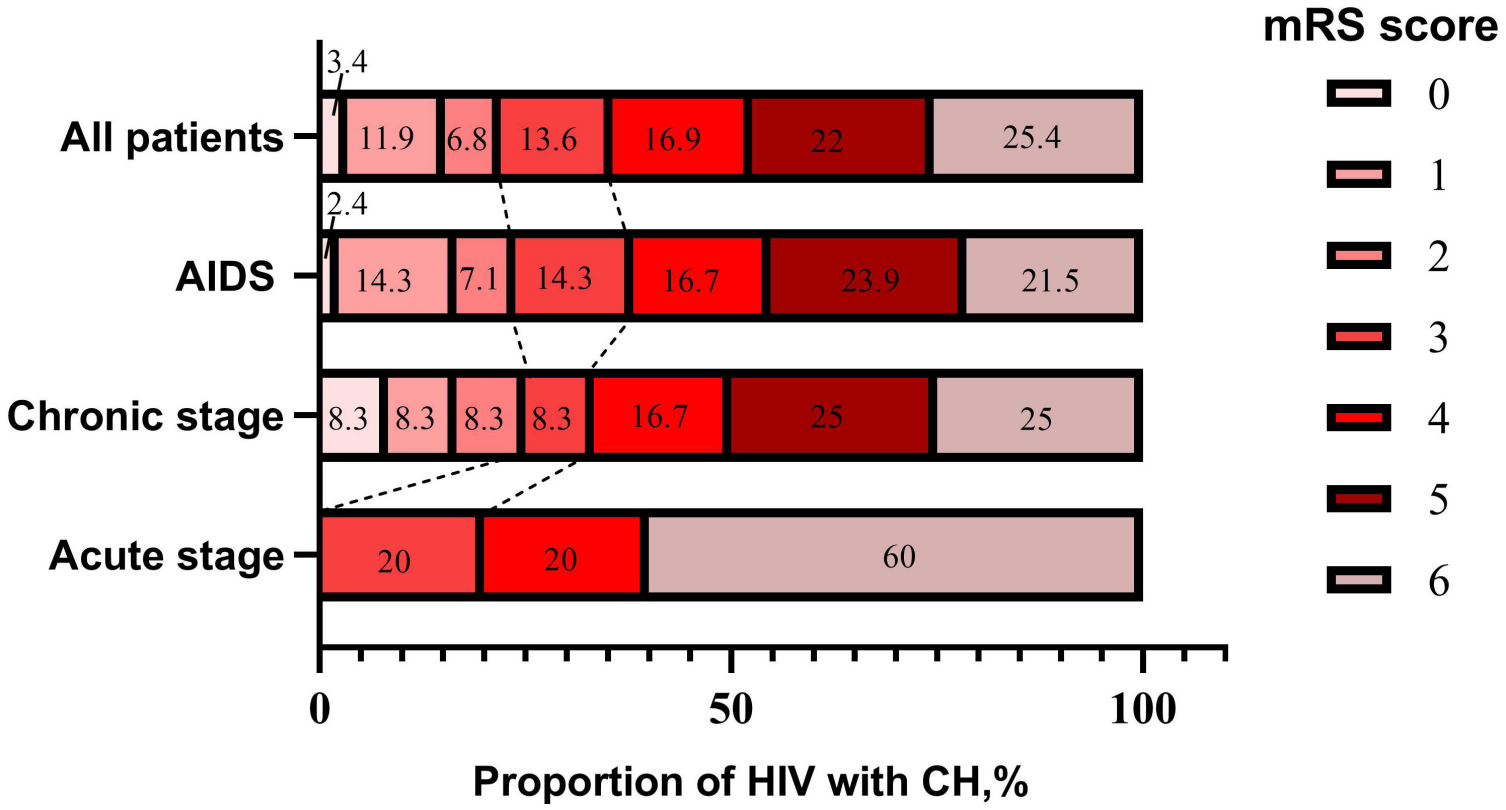
Stacked bars of mRS score in HIV-infected ICH patients at discharge.

Generally, among all patients with ICH who were HIV-infected, 22.1% had a good outcome (mRS 0-2), 77.9% had a poor outcome (mRS 3-6), and 25.4% died. Moreover, HIV-infected ICH patients had worse outcomes at discharge as the duration of HIV-infected infection increased. (**Figure 4**).

### Univariate analysis and multivariate binary logistic regression analysis

Before PSM, a comparison between 59 HIV-infected ICH patients and 180 HIV-infected without ICH patients identified seven risk factors for ICH: drug abuse-related HIV-infected, hypertension, elevated white blood cell count (WBC), elevated red blood cell count (RBC), prolonged PT, elevated serum TG levels and decreased CD4+ cell count (**Table 2**).

**Table 2:** Patients’ profiles.

Variable	Before the propensity score matching		*p-*value	After the propensity score matching		*p*-value
	HIV with ICH (n = 59)	HIV without ICH (n = 180)		HIV with ICH (n = 59)	HIV without ICH (n = 59)	
Age (year)	54.5 (48.0-63.0)	55.0 (47.0-67.0)	0.604^b^	54 (47-67)	51.0 (45-67)	0.651^b^
Male (%)	46 (78.0)	136 (75.6)	0.706^c^	46 (78)	44 (74.6)	0.665^c^
Route of transmission						
General behaviors n (%)	38 (64.4)	128 (71.1)	0.332^c^	38 (64.4)	42 (71.2)	0.431^c^
Drug abuse n (%)	14 (23.7)	12 (6.7)	< 0.001^c^	14 (23.7)	2 (3.4)	0.001^c^
Uncertain n (%)	7 (11.9)	40 (22.2)	0.082^c^	7 (11.9)	15 (25.4)	0.059^c^
Stages of HIV						
Acute stage (%)	5 (8.5)	21 (11.7)	0.456^c^	5 (8.5)	5 (8.5)	0.974^c^
Chronic stage (%)	12 (20.3)	47 (26.1)		12 (20.3)	13 (22.0)	
AIDS (%)	42 (71.2)	112 (62.2)		42 (71.2)	41 (69.5)	
Vascular risk factors						
Hypertension (%)	30 (50.8)	21 (11.7)	< 0.001^c^	31 (52.5)	22(37.3)	0.096^c^
Diabetes (%)	6 (10.2)	8 (4.4)	0.104^c^	6 (10.2)	1 (1.7)	0.051^c^
Current smoker (%)	16 (27.1)	59 (32.8)	0.416^c^	16 (27.1)	14 (23.7)	0.672^c^
Alcohol consumption (%)	14 (23.7)	42 (23.3)	0.950^c^	14 (23.7)	11 (18.6)	0.499^c^
HAART (%)	34 (57.6)	79 (43.9)	0.360^c^	34 (57.6)	23 (39.0)	0.043^c^
Blood tests						
WBC (10^9^/L)	9.0 (4.7-11.6)	5.5 (3.5-7.3)	< 0.001^b^	7.5 (4.7-11.7)	5.1 (3.4-7.2)	< 0.001^b^
RBC (10^12^/L)	3.71 (3.3-4.2)	3.3 (2.7-4.0)	0.009^b^	3.7 (3.2-4.2)	3.1 (2.5-3.9)	0.006^b^
PLT (10^9^/L)	175.5 ± 106.0	191.04 ± 105.9	0.328^a^	189.0 (73.0-240.0)	201.0 (108.0-254.0)	0.693^b^
PNC (%)	72.8 ± 14.1	68.4 (55.5-83.2)	0.143^b^	74.2 (60.8-84.7)	66.5 (54.5-81.7)	0.019^b^
PT (s)	17.0 ± 4.6	13.1 ± 2.1	< 0.001^a^	15.2 (13.8-19.3)	13.1 (11.6-14.4)	< 0.001^b^
INR	1.0 (0.9-1.2)	1.0 (0.9-1.2)	0.578^b^	1.0 (0.9-1.2)	1.0 (0.9-1.2)	0.509^b^
PTA (s)	98.0 (80.0-112.0)	97.5 (79.3-120.3)	0.969^b^	98.0 (80.0-112.0)	99.2 (81.0-120.2)	0.643^b^
APTT (s)	37.6 ± 24.0	33.9 ± 9.2	0.303^a^	33.7 (30.4-36.6)	31.1 (27.9-37.3)	0.130^b^
Triglyceride (mmol/l)	3.2 ± 1.6	1.8 ± 1.6	< 0.001^a^	2.9 (1.7-4.4)	1.4 (1.0-2.5)	< 0.001^b^
LDL (mmol/l)	2.3 ± 1.2	2.2 (1.4-2.8)	0.578^b^	2.3 (1.4-2.9)	2.2 (1.4-2.7)	0.518^b^
Immunity indicator						
CD4^+^ (cells/mm^3^)	195.0 (103.0-417.0)	101.5 (28.3-225.5)	< 0.001^b^	195.0 (103-417.0)	107.0 (32-181)	< 0.001^b^
CD8^+^ (cells/mm^3^)	419.0 (226.0-782.5)	430.5 (231.3-791.3)	0.988^b^	449.0 (224.0-672.3)	450.0 (250.0-866.0)	0.887^b^
Complications						
Syphilis n (%)	10 (16.9)	34 (18.9)	0.739^c^	10 (16.9)	9 (15.3)	0.802^c^
Hepatitis C virus n (%)	9 (15.3)	15 (8.6)	0.125^c^	9 (15.3)	2 (3.4)	0.027^c^
Rubella virus n (%)	34 (57.6)	98 (54.4)	0.670^c^	34 (57.6)	35 (59.3)	0.852^c^
Toxoplasma n (%)	4 (6.8)	17 (9.4)	0.530^c^	4 (6.8)	4 (6.8)	1.000 ^c^
Cytomegalovirus n (%)	36 (61.0)	115 (63.9)	0.691^c^	36 (61.0)	40 (67.8)	0.442^c^
Herpes simplex virus n (%)	35 (59.3)	107 (59.4)	0.987^c^	35 (59.3)	36 (61)	0.851^c^
Tuberculosis n (%)	11 (18.6)	48 (26.7)	0.215^c^	11 (18.6)	12 (20.3)	0.816^c^

Values are expressed as the mean ± SD, median (lower quartile upper quartile), or n (%); percentages are rounded to the nearest decimal point and thus may not add up to 100.

^a^Two independent samples t-test; ^b^Mann–Whitney U test; ^c^Chi-square test or Fisher’s exact test.

HIV, human immunodeficiency virus; ICH, intracerebral hemorrhage; HAART, Highly Active Antiretroviral Therapy; WBC, white blood cell count; RBC, red blood cell count; PNC, percentage of neutrophil count; PLT, platelet; PT, prothrombin time; INR: International normalized ratio; APTT, activated partial thromboplastin time; Cr, creatinine; LDL, low-density lipoprotein; CD4, CD4+ T cell count; CD8, CD8+ T cell count.

Furthermore, univariate analysis after PSM revealed nine risk factors for ICH: drug abuse-related HIV-infected, HAART usage, elevated WBC, elevated RBC, elevated neutrophil count percentage, prolonged PT, elevated serum TG levels, and decreased CD4+ cell count, as well as an increasing incidence of hepatitis C virus contraction (**Table 2**).

Multivariate binary logistic analysis revealed that drug abuse-related HIV (*P*=0.024, odds ratio (OR) 8.107, 95% confidence interval [CI], [1.310-50.155]), prolonged PT (*P*= < 0.001, OR 1.781, 95% CI, [1.328-2.388]), elevated serum TG levels (*P*=0.048, OR 1.423, 95% CI, [1.002-2.021]) independently contributed to the risk of ICH in HIV-infected patients (**Table 3**).

**Table 3:** Patients’ profiles for multivariate logistic regression analysis.

Variable	After the propensity score matching		
	β Value	*P*-Value	OR (95%CI)
Drug abuse n (%)	2.093	0.024	8.107 (1.310-50.155)
HAART (%)	0.12	0.839	1.127 (0.357-3.562)
WBC (10^9^/L)	0.058	0.446	1.06 (0.912-1.232)
RBC (10^9^/L)	0.458	0.155	1.581 (0.841-2.971)
PNC (%)	0.011	0.641	1.011 (0.966-1.057)
PT (s)	0.577	< 0.001	1.781 (1.328-2.388)
Triglyceride (mmol/l)	0.353	0.048	1.423 (1.002-2.021)
CD4+ (mm^3^)	0.003	0.092	1.003 (1.000-1.006)
Hepatitis C virus n (%)	1.347	0.182	3.846 (0.531-27.853)

HAART, Highly Active Antiretroviral Therapy; WBC, white blood cell count; RBC, red blood cell count; PNC, percentage of neutrophil count; PT, prothrombin time; CD4, CD4+ T cell count.

### Receiver operating characteristic curves

**Figure 5:**
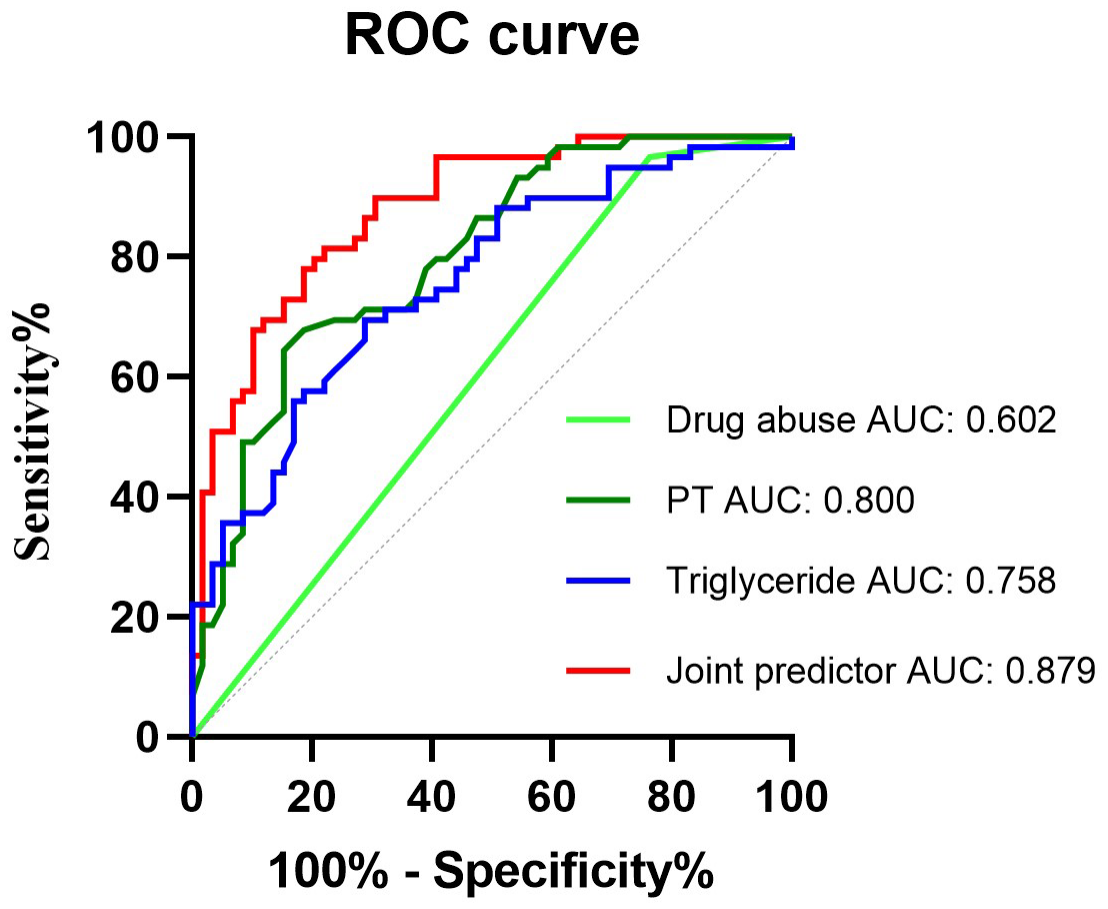
Receiver operating characteristic curves obtained with different discriminatory models for predicting the occurrence of HIV-infected ICH. The combined predictive factor showed the most significant AUC of 0.879 (95% confidence interval [CI] 0.819–0.939, *P* < 0.001). The optimum cut-off point was 0.426. At this threshold value, the sensitivity was 78%, and the specificity was 81.4%; CI, confidence interval; ROC, receiver operator characteristic; AUC, the area under the curve.

ROC was used to assess the predictive value of drug abuse-related HIV, prolonged PT, elevated serum TG levels, and the combined predictor (comprising drug abuse-related HIV, prolonged PT, and elevated serum TG levels) for the development of ICH in HIV-infected patients. The ROC curve indicated that the combined predictor exhibited the highest AUC, measuring 0.879 (95% CI 0.819–0.939, *P* < 0.001), signifying excellent overall diagnostic accuracy. The optimal diagnostic threshold value for the combined predictor, as determined by ROC analysis, was 0.426, with a sensitivity of 78.0% and specificity of 81.4% (**Figure 5**).

## Discussion

Patients with HIV-infected were at an elevated risk of ICH. The present study aimed to comprehensively explore the clinical features and independent risk factors of ICH in HIV-infected patients.

### Clinical features of HIV-infected ICH patients

In the present study, most HIV-infected ICH patients were middle-aged (35 to 50 years of age) and elderly (60 years of age and over) males. Similarly, Patel reported that most HIV-infected ICH patients had a mean age of 47 years (Patel et al., 2021), suggesting that middle-aged and older men with HIV-infected have a higher propensity to develop ICH than women of similar age. Moreover, in the present study, five patients were hospitalized due to the first-ever ICH, and the HIV infection was first established. As previous studies also showed that some patients with first-ever ICH without cerebrovascular risk factors were first found to have HIV-infected infection during hospitalization (Roquer et al., 1998), indicating that ICH may serve as the initial manifestation in some HIV-infected patients, it was suggested that screening for the cause of ICH without risk factors should include HIV-infected infection.

In the present study, it was found that an overwhelming majority of HIV-infected patients with ICH were in the AIDS stage, strongly suggesting that ICH in these patients was directly or indirectly related to HIV-infected. A population-based cohort study in 2013, including 7,053 patients with HIV-infected and 27,681 without, found that AIDS-stage patients were at a significantly higher risk of ICH (Durand et al., 2013). Another study found that HIV significantly increased the risk of ICH over three times, particularly during the advanced stages of the disease (Behrouz et al., 2016). These findings further suggested that ICH in patients with HIV infection was caused directly or indirectly by HIV. Moreover, in the present study, most HIV-infected ICH patients developed one or more infections, such as syphilis, tuberculosis, toxoplasma, and hepatitis C viral infection, which can have neurological manifestations and complicate the clinical course of HIV-infected infection. Previous studies also found that opportunistic infections, including neurosyphilis, toxoplasmosis, and cytomegalovirus, were found to be expected in HIV-infected ICH patients in an advanced stage (Patel et al., 2021). Although the relationship between HIV-infected ICH patients and opportunistic infections remains not fully understood, it is reasonable that clinicians should pay more attention to controlling the complications of the infection in patients with HIV-infected. Additionally, most HIV-infected ICH patients had a poor short prognosis, which was similar to previous studies (Roquer et al., 1998; Patel et al., 2021), indicating that it was a challenge to prevent and treat ICH in patients with HIV-infected, especially for those in an advanced stage.

### The risk factors of ICH in HIV-infected patients

In the present study, PSM was employed to eliminate the confounding effects of conventional vascular risk factors, aiming to identify independent risk factors of ICH for the patients with HIV-infected. It was revealed that drug abuse-related HIV-infected was independently associated with ICH in HIV-infected patients. Previous research has indicated a strong correlation between drug abuse in HIV-infected patients with ICH, including the misuse of cocaine, amphetamine, and ecstasy (Pozzi et al., 2008; Rasmussen et al., 2011; Durand et al., 2013; Thakur et al., 2016). Although the precise mechanism of ICH induced by drug abuse remained unclear (Treadwell and Robinson, 2007), it was supposed that multiple factors were involved, including vasospasm, cerebral vasculitis, cardioembolism, and hypertensive surges associated with altered cerebral autoregulation (Green et al., 1990). Therefore, drug abuse may play a contributing role in the occurrence of ICH in patients with HIV infection through one or more of the mechanisms mentioned above. These findings highlighted that some unique strategies in prevention and management should be taken for drug abuse-associated ICH in patients with HIV-infected.

In the present study, prolonged PT was another independent risk factor of ICH in patients with HIV infection. As it was demonstrated that ICH in patients with HIV-infected was related to coagulopathy in a cohort and nested case-control study involving 7,053 HIV-infected-positive and 27,681 HIV-infected-negative individuals (Durand et al., 2013), the finding in the present study further substantiated the correlation between prolonged PT and ICH in HIV-infected patients. It should be noted, however, that protracted PT has also been identified as a risk factor for ICH in non-HIV-infected patients (Zhang et al., 2017). This suggests that coagulation dysfunction may be one of the common pathological mechanisms of ICH in both HIV-infected and non-HIV-infected patients. Given this, future studies should focus on developing more accurate predictors to individually assess and predict ICH risk in HIV-infected patients to understand the complex mechanisms of ICH in HIV-infected patients and provide more precise guidance for clinical intervention.

In the present study, elevated SERUM TG levels were identified as an independent risk factor of ICH for patients with HIV infection. Similar findings were shown in previous studies in common populations. Firstly, elevated SERUM TG levels were associated with deep or infratentorial and any microbleeds but not with lobar microbleeds (Lei et al., 2014). Secondly, elevated serum TG levels exhibited a significant increase in a cross-sectional study with 500 ICH patients, and the linear regression and correlation analysis showed that elevated serum TG levels gradually increased the volumes of ICH enlarger (Zhou et al., 2003). Thirdly, a prospective cohort study with 11,699 participants found that elevated serum TG/HDL-C levels were significantly associated with an increased risk of ICH in individuals with a healthy body mass index (Sato et al., 2022). To sum up, it was suggested that elevated serum TG levels could be regarded as a potential risk of ICH in the standard population. However, although previous studies showed that elevated serum TG level was a common dyslipidemia in HIV-infected patients (Hsue and Waters, 2019; Waters and Hsue, 2019), elevated serum TG levels were associated with an increased risk of atherosclerosis and vascular inflammation in HIV-infected patients (Raposeiras-Roubin et al., 2021; Toth, 2021). The relationship between elevated serum TG levels and ICH in patients with HIV-infected remained unclear. However, the findings in the present study further suggested that elevated serum TG level could be a risk of ICH for patients with HIV-infected, indicating that actively controlling the serum TG level may be adequate to prevent ICH in patients with HIV-infected.

In fact, in the present study, considering the development of ICH in patients with HIV-infected may be the combined effect of drug abuse-related HIV-infected, prolonged PT, and elevated serum TG levels, a combined predictor comprising the three factors mentioned above was calculated. The ROC curve showed that the combined predictor exhibited the highest AUC, with an optimal diagnostic threshold value of 0.426. This suggested that the combined predictor could potentially serve as a biomarker for identifying HIV-infected patients at high risk of ICH and facilitate clinicians in implementing appropriate preventive measures accordingly.

There were certain limitations of the study. Firstly, our study had a relatively small sample size due to stringent inclusion and exclusion criteria. Although the survey employed retrospective comparisons using PSM analysis to mitigate bias in patient selection, potential unobserved confounding factors may still exist. Further, more extensive and diverse studies are needed to validate our findings. Furthermore, data collection for this study spanned a 20-year period during which standard treatment protocols may have significantly evolved; however, our research could not comprehensively capture these changes in treatment approaches. To adequately investigate the underlying mechanisms of ICH in HIV-infected patients, further studies are warranted.

## Conclusion

In summary, it was found that factors including drug abuse-related HIV-infected, prolonged PT, and elevated serum TG levels were the independent risk factors of ICH in HIV-infected patients. The combined predictor may serve as a valuable biomarker of predictor for ICH in patients with HIV-infected. However, the current study’s findings need to be validated by further scientific research.

## Data Availability

The original contributions presented in the study are included in the article/supplementary material. Further inquiries can be directed to the corresponding author.
